# Inhibition of Cytochrome P450 3A in Rat Liver by the Diorganotin (IV) Compound di-*n*-Butyl-di-(4-chlorobenzo-hydroxamato)tin (IV) and Its Probable Mechanism 

**DOI:** 10.3390/molecules170910994

**Published:** 2012-09-12

**Authors:** Yunxia Zhang, Yunlan Li, Qingshan Li

**Affiliations:** School of Pharmaceutical Science, Shanxi Medical University, 56 Xinjian South Road, Taiyuan 030001, Shanxi, China; Email: zyxia.cafe@163.com

**Keywords:** cytochrome P450 3A (CYP3A), di-*n*-butyl-di-(4-chlorobenzohydroxamato)tin (IV) (DBDCT), inhibition, rat

## Abstract

The specific aims of this study were to evaluate the inhibition effect on CYP3A of di-*n*-butyl-di-(4-chlorobenzohydroxamato)tin (IV) (DBDCT), a tin-based complex with high antitumor activity, and the probable mechanism(s) of this action. Adult male SD rats were treated separately with natural saline (NS), lipopolysaccharide (LPS, 5 mg/kg), DBDCT (1.25, 2.5 and 5.0 mg/kg) intraperitoneally for 2 days after induction of CYP3A with dexamethasone (DEX, 100 mg/kg) for 4 days. Western blot analysis and fluorescent quantitation PCR (FQ-PCR) were conducted to determine the changes in expression of CYP3A, PXR, CAR and RXR. The biological accumulation of DBDCT and total Sn were determined by high-performance liquid chromatography (HPLC) and atomic fluorescence spectrometry (AFS). CYP450 content and CYP3A activities were significantly inhibited (*p* < 0.05) in DBDCT-treated rats compared with the control group, as was the expression of CYP3A (*p* < 0.05) at both protein and mRNA levels. In DBDCT-treated groups, the expression of PXR protein and mRNA increased, while the expression of CAR decreased. The biological accumulation of DBDCT and Sn in rat livers treated with DBDCT was high. The accumulation of DBDCT and Sn due to the inhibition of CYP3A may be involved in the mechanism of toxicity of DBDCT in rat liver.

## 1. Introduction

Significant attention has been focused on organotin(IV) compounds in recent years, following reports on the antitumor activities of dialkyltin(IV) derivatives [1,2,3,4]. We have reported [5,6,7] on the synthesis, activities and mechanisms of action of several series of diorganotin(IV)/arylhydroxamates after a series of researches on diorganotin(IV) complexes with arylhydroxamate ligands, and found that they had strong antitumor activity. Recently, a new patented diorganotin(IV) arylhydroxamate, di-*n*-butyl-di-(4-chlorobenzhydroxamato)tin (IV) (DBDCT) was synthesized [6] in our laboratory, which exhibited strong *in vitro* and *in vivo* antitumor activity, and its activity, in some cases, was close to, or even higher than that of cisplatin. 

Experiments on the metabolism of DBDCT with different enzyme sources and metabolic inhibition tests have been conducted *in vitro*. These experiments showed that of the CYP450 isoforms, CYP3A plays a leading role in catalytic DBDCT metabolism, and CYP2C9 may be partly involved. Substrate test results also showed that DBDCT had a strong inhibitory effect on CYP3A and no significant effect on CYP1A, CYP2C9 and CYP2C19 [8]. CYP450s have been shown to be critical in the metabolism of xenobiotics including the vast majority of clinically used drugs, environmental procarcinogens and toxins [9,10]. It has been suggested that CYP3A, the most important CYP450 in the liver, plays a major role in the biotransformation of many drugs, and is responsible for the oxidative metabolism of more than 60% of all pharmaceuticals [11,12]. The expression of CYP3A is affected by chemical inducers, nutritional conditions, growth factors and inflammatory mediators through nuclear receptor signaling pathways. Two nuclear receptors, pregnane X receptor (PXR) and constitutive androstane receptor (CAR), mediated the effects of xenobiotics and therapeutic drugs on the regulation of CYP3A [13]. The literature shows that PXR and CAR regulate the expression of target genes, such as CYP3A, by binding with its obligate partner retinoid receptor (RXR) to form a heterodimer which has high DNA binding affinity [14,15].

In the present study, photomicrographs of rat liver tissues using HE staining in acute toxicity tests demonstrated severe liver lesions. The probable reasons for these lesions include reactive oxygen species (ROS) generation, apoptosis (data not shown) and the accumulation of DBDCT and Sn. The function of CYP3A was significantly inhibited by DBDCT, however, the inhibitory mechanisms are unclear. Thus, the objectives of this study were to provide molecular evidence of the repression of CYP3A expression by DBDCT at the protein and mRNA levels in rat livers, and to predict the roles of PXR and CAR in the down-regulation of CYP3A. The accumulation of Sn in rat liver was also assessed to determine the probable mechanism of *in vivo* DBDCT toxicity.

## 2. Results and Discussion

### 2.1. DBDCT Reduced the Liver Weight Ratio, Decreased Microsomal Protein and CYP450 Content in Rat Liver

Male SD rats were treated with 1.25, 2.5 and 5.0 mg/kg DBDCT i.p. once daily for 2 days after being induced of CYP3A with DEX (100 mg/kg, i.p.) for 4 days. Blank control animals were treated with saline after DEX induction. Each value represented the mean ± SD for six animals. 

Rat liver weight ratio and CYP450 content in the LPS group (5.0 mg/kg) were markedly decreased (*p* < 0.01) compared with those in the control group, which demonstrated that our models were successfully established. The liver weight ratio was the relative value of liver weight compared with rat body weight. The body weight of rats treated with DBDCT did not change significantly (*p* > 0.05), however, the rat liver weight ratio decreased significantly compared with that in the control group (Table 1). Compared with the control group, the relative liver ratio of rats treated with DBDCT at doses of 1.25, 2.5 and 5.0 mg/kg decreased by 7.9% (*p* > 0.05), 22.7% (*p* < 0.001) and 20.8% (*p* < 0.001), respectively. Liver microsomal protein concentration in the 2.5 and 5.0 mg/kg DBDCT-treated groups and CYP450 content were significantly reduced (*p* < 0.01) compared with those in the control group (Table 1). 

**Table 1:** The influence of DBDCT on rats liver.

Parameters	Control	LPS group	DBDCT (mg/kg)		
			1.25 (L)	2.50 (M)	5.0 (H)
Rat weight (g)	210 ± 14	200 ± 6	207 ± 9	203 ± 12	208 ± 15
Liver weight ratio (g/g)%	6.31 ± 0.46	4.97 ± 0.34 **	5.80 ± 0.40	4.88 ± 0.53 **	4.99 ± 0.32 *
Protein (mg·g^−1^liver)	16.40 ± 2.74	14.02 ± 3.16	17.06 ± 2.27	11.02 ± 1.82 **	11.63 ± 1.39 **
CYP450 (nmol·mL^−1^)	1.12 ± 0.11	0.52 ± 0.11 **	0.66 ± 0.08 **	0.56 ± 0.07 **	0.53 ± 0.05 **

Notice: * *p* < 0.05, ** *p* < 0.01, compared with control group.

### 2.2. DBDCT Decreased the Activity of CYP3A

**Figure 1:**
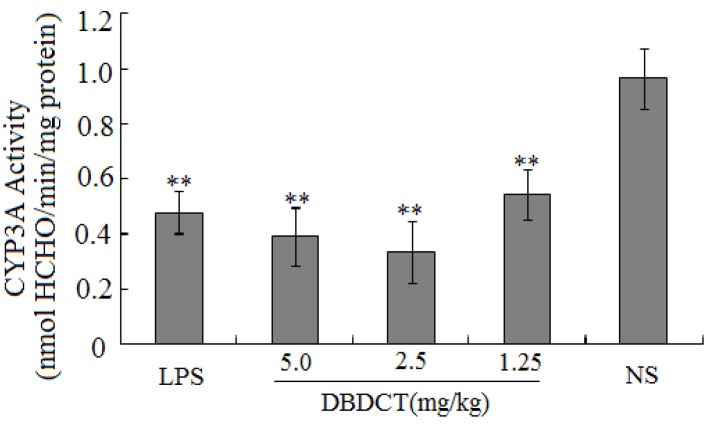
The influence of DBDCT on the activity of CYP3A. * *p* < 0.05, ** *p* < 0.01, compared with blank control group.

CYP3A activity was assayed by the method of Nash [16]. Compared with the control group, rats treated with DBDCT had low CYP3A activity (*p* < 0.01), as shown in Figure 1. In addition, CYP3A activity in the 2.5 or 5.0 mg/kg groups was lower than that in the 1.25 mg/kg group. 

### 2.3. DBDCT Reduced Liver Microsomal Proteins and mRNA Expression of CYP 3A1/2 in Rats

**Figure 2:**
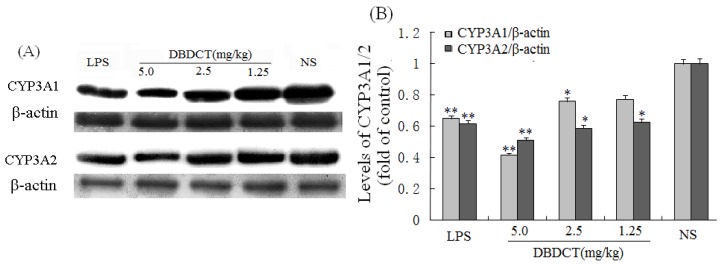
Repression effect of DBDCT on CYP3A1/2 protein expression in rat liver microsomes. (**A**) Immunoblots of CYP3A1/2 from rat liver microsomes. Microsomal proteins from blank controls and DBDCT-treated rats were subjected to protein blot analyses in which antibodies against CYP3A1/2 and β-actin were used to probe for immunorelated proteins as described in text; (**B**) Relative protein expression level of CYP3A1/2 in rat liver. The results were expressed as a ratio to the expression levels of the reference protein β-actin. Each value represents the mean ± SD for six animals; * *p* < 0.05, ** *p* < 0.01, compared with control group.

Immunoblotting studies were conducted to investigate the effects of DBDCT on CYP3A1 and CYP3A2 protein level in rat liver microsomes. Liver microsomal proteins were subjected to gel electrophoresis and immunoblot analysis using antibodies against CYP3A1 and CYP3A2 (Figure 2). The results of immunoblot analysis showed that the expression of CYP3A1 and CYP3A2 proteins in rat liver was markedly decreased (*p* < 0.05) following treatment with DBDCT at different doses, the strongest inhibitory effect was demonstrated in the DBDCT 5.0 mg/kg dose group compared with the control group, which was consistent with CYP450 content. The CYP3A1 expression levels of microsomal proteins in rats treated with 1.25, 2.5 and 5.0 mg/kg DBDCT decreased by 13.7%, 30.4% (*p* < 0.05) and 53.5% (*p* < 0.001), respectively, while CYP3A2 expression decreased by 30.9% (*p* < 0.05), 32.2% (*p* < 0.05) and 54.4% (*p* < 0.001), respectively. 

**Figure 3:**
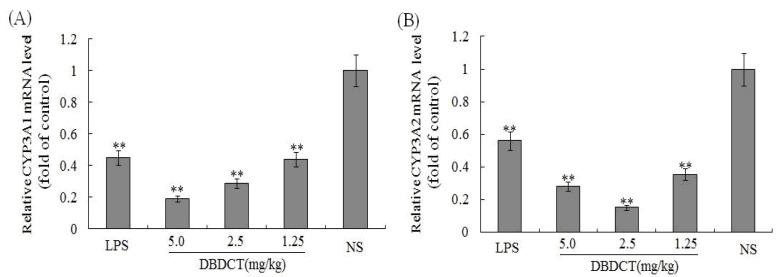
Relative mRNA expression level of CYP3A1/2 in rat liver. (**A**) and (**B**), separately showed the relative mRNA expression level of CYP3A1/2 in rat liver. The results were expressed as a ratio to the mRNA levels of the reference gene GAPDH. Each value represents the mean ± SD for six animals; * *p* < 0.05; ** *p* < 0.01, compared with the control group.

To determine whether the CYP3A1/2 mRNA levels in rat liver microsomes were decreased by DBDCT as the protein decreased, FQ-PCR was performed. As shown in Figure 3, CYP3A1 in rats treated with LPS decreased by 45.1% (*p* < 0.01) compared with that in the control group. CYP3A1 protein expression in rats treated with DBDCT was significantly reduced in a dose- and time-dependent manner. The expression of CYP3A2 in the LPS group was also significantly depressed by 56.3% (*p* < 0.01). Although the CYP3A2 protein expression in rats treated with DBDCT in all three dose groups was low, this expression was not dose- or time-dependent. The 2.5 mg/kg group had a lower CYP3A2 expression level (decreased to 15.1%, *p* < 0.01) than the 5.0 mg/kg DBDCT group (decreased to 28.3%, *p* < 0.01) and the 1.25 mg/kg DBDCT group (decreased to 35.4%, *p*< 0.01) compared to that in the control group. These results were in agreement with the protein concentration and CYP3A activity findings. 

### 2.4. Effects of DBDCT on Nuclear Proteins and mRNA Expression of PXR, CAR and RXR in Rat Liver

**Figure 4:**
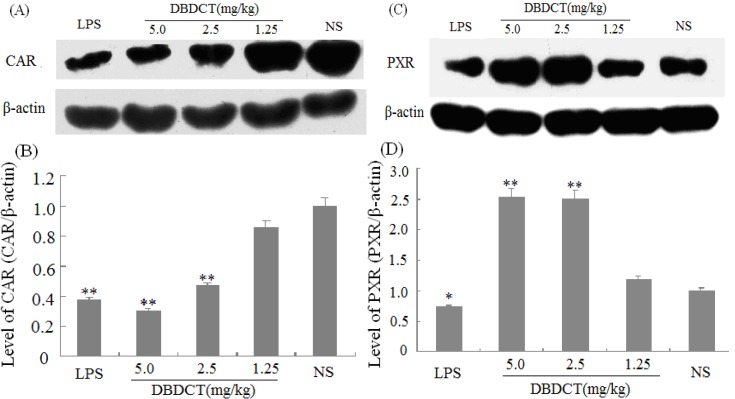
Effect of DBDCT on nuclear receptor protein expression in rat liver. (**A**) Immunoblots of CAR from rat liver nuclear protein; (**B**) Relative protein expression level of CAR in rat liver; (**C**) Immunoblots of PXR from rat liver nuclear protein; (**D**) Relative protein expression level of PXR in rat liver. The relative protein expression levels were expressed as a ratio to the expression levels of the reference protein β-actin. Each value represents the mean ± SD for six animals; * *p* < 0.05; ** *p* < 0.01, compared with control group.

**Figure 5:**
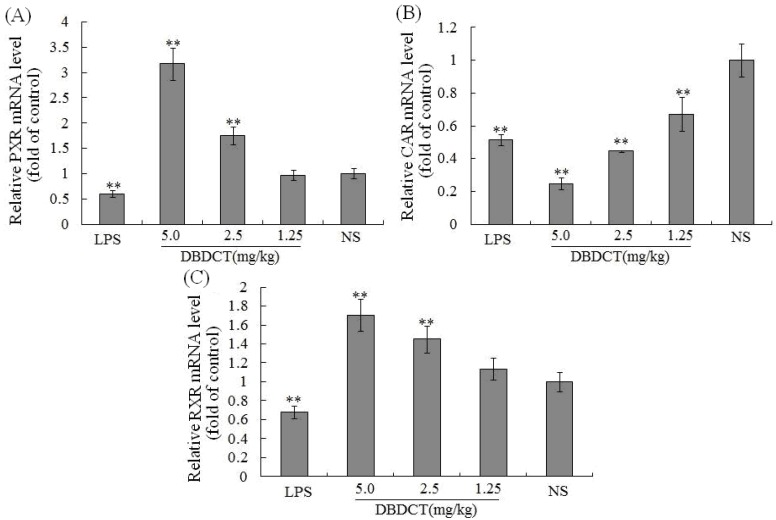
Effect of DBDCT on nuclear receptor mRNA level in rat liver. (**A**), (**B**) and (**C**), separately showed the relative mRNA expression level of PXR, CAR and RXR in rat liver. The FQ-PCR data were calculated using the 2^−ΔΔCt^ method normalized to the individual internal control (GAPDH) level. Each value represents the mean ± SD for six animals; * *p* < 0.05, ** *p* < 0.01, compared with control groups.

We evaluated the effects of DBDCT on the expression of nuclear receptors PXR, CAR, and RXR using immunoblot and FQ-PCR analysis. As shown in Figure 4A,B, the nuclear protein expression level of CAR was markedly decreased (*p* < 0.01) after treatment with DBDCT at the dose of 2.5 and 5.0 mg/kg. It can be seen from Figure 4C,D that the PXR protein expression level was significantly increased (*p* < 0.05) in the DBDCT 2.5 mg/kg and 5.0 mg/kg groups compared with that in the control group, and significant up-regulation of PXR mRNA expression level was also detected (Figure 5A) which increased to 175.0% (*p* < 0.01) in the DBDCT 2.5 mg/kg group and to 317.7% in the 5.0 mg/kg group (*p* < 0.01) compared with that in the control group. In addition, CAR mRNA level was reduced dose-dependently in the three groups (Figure 5B). RXR mRNA level was also detected using FQ-PCR. These results (Figure 5C) showed that the mRNA level of RXR decreased in rats treated with LPS (68.0%, *p* < 0.01), and increased in DBDCT-treated rats at the dosage of 1.25 (113.7%, *p* > 0.05), 2.5 (145.0%, *p* < 0.01) and 5.0 (170.7%, *p* < 0.01) mg/kg. 

### 2.5. Histopathology

**Figure 6:**
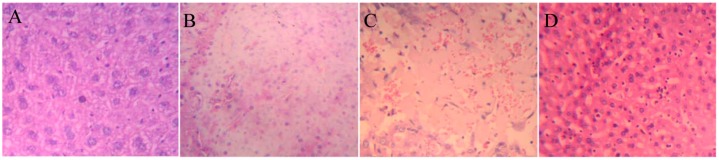
Representative photomicrographs of the liver sections from DBDCT acute toxicity testing stained with hematoxylin and eosin (magnification 20×). (**A**) liver section of control group, a normal histology with regular morphology of the liver tissue and well-organized hepatic cells; (**B**) liver section of DBDCT 60 mg/kg group, acute necrosis of liver cells; (**C**) liver section of DBDCT 48 mg/kg group, focal necrosis of liver cells; (**D**) liver section of DBDCT 48 mg/kg group, Kupffer cells hyperplasia.

To verify the liver toxicity of DBDCT, histopathological examination of liver tissues was performed. Microscopic observations revealed a normal histology with regular morphology of the liver tissue and well-organized hepatic cells in the control group (Figure 6Aacute necrosis (Figure 6B, DBDCT 60 mg/kg), focal necrosis (Figure 6C DBDCT 48 mg/kg D, DBDCT 48 mg/kg) of liver cells. 

### 2.6. Biological Accumulation of DBDCT in Rat Liver

DBDCT (5 mg/kg) was administered by a single intravenous injection, and control rats received saline. Blood samples were collected at designated time intervals (3, 10, 50 and 120 min after injection of DBDCT), and rats were then sacrificed to collect organs. Plasma samples were obtained immediately from the blood samples by centrifugation at 4,000 × *g* for 10 min at 4 °C. 

**Figure 7:**
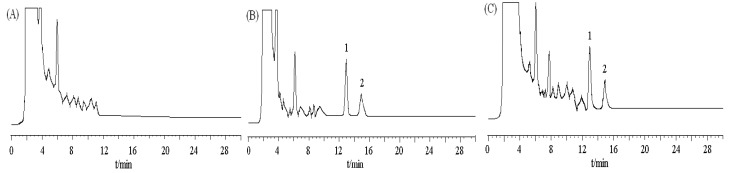
Chromatograms of DBDCT and internal standard in rat liver. (**A**) blank tissue; (**B**) blank tissue spiked with DBDCT (0.6 μg/mL)and acetanilide (I.S., 2.5 μg/mL); (**C**) 1iver tissue sample at 50 min after intravenous administration of DBDCT at a dose 5 mg/kg; peak 1 internal standard (acetanilide); peak 2 DBDCT.

**Figure 8:**
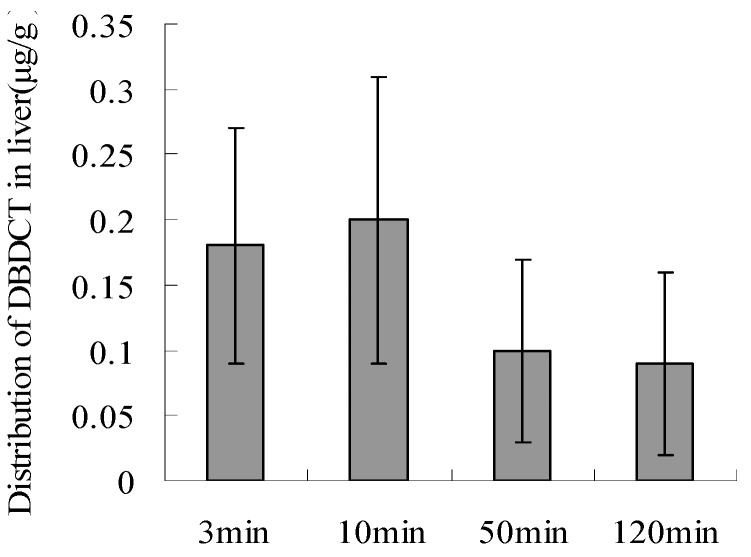
The biological accumulation of DBDCT in rat liver as detected by HPLC.

Approximately 50 min after injection, DBDCT was distributed in the main organs, and about 120 min later, DBDCT was detected in a few organs(data is not shown), especially in the liver. This demonstrated that DBDCT accumulated in rat liver (Figure 7 and Figure 8). 

**Figure 9:**
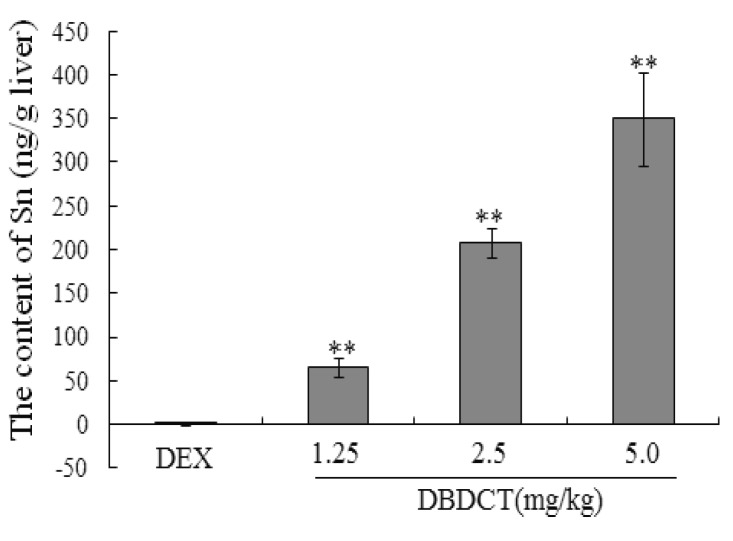
The biological accumulation of Sn in rat liver. Male SD rats were treated with 1.25, 2.5 and 5.0 mg/kg DBDCT once daily for 2 days after induction with 100 mg/kg DEX for 4 days. Blank control animals were treated with saline and the positive control animals with 5 mg/kg LPS after DEX induced. Each value presented the mean ± SD for six animals. About 0.5 mg rat liver was digested with 0.2 mL sulfuric acid and 2.0 mL nitric acid in glass tubes for one night at room temperature. On the second day, the tubes were heated to volatilize all the nitric acid until white smoke was generated. The solution left in the tubes was diluted to 10 mL for tested. Each value represents the mean ± SD for six animals; * *p* < 0.05; ** *p* < 0.01, compared with control group.

In order to determine whether biological accumulation of Sn occurred in rat liver to predict the probable mechanism of *in vivo* DBDCT toxicity, atomic fluorescence spectrometry was used to detect Sn content in rat liver. As shown in Figure 9, Sn in rat liver was accumulated in a dose-dependent manner. 

**Figure 10:**
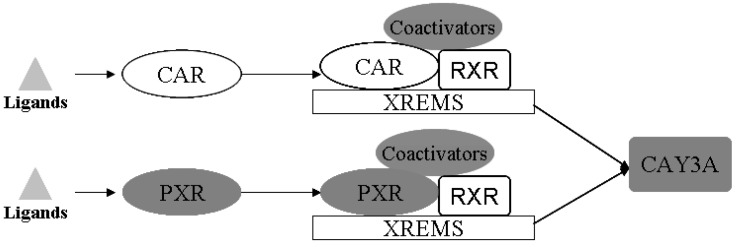
Schematic illustration of the activation mechanisms and target genes of CAR and PXR. PXR and CAR can be activated by ligand binding. CAR and PXR shared target genes both through binding with RXR, and mediated the regulation of expression of CYP3A (modified from [17]).

Drug-metabolizing enzymes (DMEs) play pivotal roles in the disposition and detoxification of numerous exogenous and endogenous chemicals. To accommodate chemical challenges, the expression of many DMEs is up-regulated by a group of ligand-activated transcription factors namely the nuclear receptors (NRs). The most important NRs in xenobiotic metabolism and clearance are the pregnane X receptor (PXR, NR1I2) and the constitutive androstane receptor (CAR, NR1I3) [17]. These two receptors mediate the effects of xenobiotics and therapeutic drugs on the regulation of CYP3A [18]. Sequence analysis showed that PXR and CAR are closely related to each other, and both require heterodimerization with RXR for high-affinity DNA binding [19,20], as shown in Figure 10. 

CYP3A is the most abundant CYP450 enzyme in the liver. It is a critical player in the metabolism of xenobiotics including the vast majority of clinically used drugs, environmental procarcinogens and toxins. Inhibition of CYP3A can decrease the metabolic rate of some prescribed drugs which are mainly metabolized by CYP3A, resulting in bioaccumulation and toxicity which are dangerous to human health. 

In the acute toxicity tests, significant damage was observed in liver sections (Figure 6). ROS generation, apoptosis and accumulation of DBDCT or Sn were probably the remote causes of this toxicity, and both ROS generation and apoptosis were confirmed (data not shown). The biological accumulation of DBDCT or Sn in the liver is usually caused by inhibition of DEMs, which is mainly mediated by PXR and CAR. 

In this study, there were several important findings. First, the liver weight ratio, protein contents and CYP450 content in rat liver microsomes were assessed to determine whether CYP450 was decreased after treatment with DBDCT (Table 1). CYP450 was found to be inhibited in rats treated with DBDCT. Second, the activity of CYP3A was determined and the results showed that the function of CYP3A was indeed inhibited (Figure 1). Rats treated with 5.0 mg/kg DBDCT had higher CYP3A activity than those treated with 2.5 mg/kg DBDCT. This may have been due to the rat partially compensating for part of the function of CYP3A when CYP3A1/2 expression was significantly decreased. Third, protein and mRNA expression were assessed to evaluate their inhibition by DBDCT in rat liver (Figure 2). The protein expression levels of CYP3A1/2 were reduced dose-dependently following exposure to DBDCT, and CYP3A1 mRNA level was also reduced (Figure 3). Fourth, PXR and CAR protein and mRNA expression levels (Figure 4 and Figure 5) were tested to determine the variance in nuclear receptor expression. Some studies have suggested that the down-regulation of CYP450 by cytokines was due to the down-regulation of PXR and CAR [21], while other studies argue that the down-regulation of P450 during inflammation does not require PXR based on the data from PXR knockout experiments in mice [22]. In the present study, the results demonstrated that PXR increased (*p* < 0.01), while CAR decreased (*p* < 0.01) after DBDCT treatment. PXR and CAR both required heterodimerization with RXR. The increase (*p* < 0.01) in RXR mRNA proved that PXR increased. It was predicted that CAR may be involved in the down-regulation of CYP3A1/2, however, it is unclear whether PXR participates in the down-regulation of PXR. Further work, such as reporter experiments, to explore the exact signaling pathway is underway. Furthermore, the accumulation of DBDCT and Sn in rat liver (Figure 8 and Figure 9) allowed us to identify possible toxic substances. The inhibition of DMEs always accompanies a broad range of drug-drug interactions, an alteration in therapeutic effect, biological accumulation and toxicity of many drugs [23]. Sn accumulation in rat liver was significant, which may have been caused by the strong inhibitory effect of CYP3A following treatment with DBDCT. The expression of CYP3A1/2 at both protein and mRNA levels was inhibited by DBDCT, as well as CYP3A enzyme activity in rat liver. These results suggest that the down-regulation of CYP3A1/2 by DBCDCT in rat liver is probably mediated by CAR. The biological accumulation of Sn due to the inhibition of CYP3A1/2 mediated by CAR and PXR may be involved in the mechanism of toxicity of DBDCT in rat liver.

## 3. Experimental

### 3.1. Chemicals and Supplies

DBDCT, synthesized by Shanxi Medical University [5], with purity over 99% by HPLC analysis, lipopolysaccharide (LPS), dexamethasone (DEX), erythromycin (ERY), nicotinamide adenine dinucleotide phosphate (NADPH) were purchased from Sigma-Aldrich (St. Louis, MO, USA). The antibodies against CYP3A1, CYP3A2 and β-actin were from Millipore (Billerica, MA, USA). The antibodies against PXR and CAR were from Santa Cruz Biotech (Santa Cruz, CA, USA) and Abcam (Abcam, Cambridge, MA, USA), respectively. The goat anti-rabbit IgG conjugated with horseradish peroxidase and eECL Western blotting kit were from CW Biotech (Beijing, China). BCA assay kit was from Beyotime Institute of Biotechnology (Shanghai, China). All primer sequences were synthesized by Sangon (Shanghai, China). RNAiso Plus, PrimeScript Reverse Transcription reagent kit (with RNA Eraser), RT primer and SYBR Premix Ex Taq^TM^ were from TaKaRa (Dalian, China). All other reagents used were of the purest grade available.

### 3.2. Animals and Treatment

Male Sprague Dawley (SD) rats weighing 200–220 g, purchased from the Laboratory Animal Center of Academy of PLA Military Medical Sciences [No. SCXK-(M)2007-004, PLA AMMS, Beijing, China], and Kunming (KM) mice (half males, weighing 18–22 g, 5–6 weeks) purchased from The Laboratory Animal Center of Hebei Province [No. SCXK(J)2008-1-003] were maintained in a climate-controlled room (poplar shavings bedding, 19–25 °C) with 12 h light/dark cycle and allowed access to a commercial rat chow (and cobalt-60 irradiation sterilization rat granulated feed) and tap water *ad libitum*. The animals were allowed to adapt to the environment for at least a week and induced by a selective CYP3A inducer (DEX, 100 mg/kg) for 4 days prior to experiment, and then, they were randomly divided into five groups, with six rats in each: blank control saline group, a selective CYP3A inhibitor LPS positive control group (5 mg/kg) and groups administered DBDCT 1.25, 2.5, 5.0 mg/kg, once daily for 2 days, respectively. The corresponding dosages were applied by intraperitoneal injection. 

### 3.3. Rat Liver Microsomes Isolation

After the last injection at the sixth day, all rats were fasted for 18 h, then sacrificed. The livers of these rats were washed and homogenized with 0.25 mol/L sucrose solution. All equipment was precooled to 4 °C. The rat liver homogenate was centrifuged at 20,000 × *g* for 20 min at 4 °C using an ultracentrifuge (Hitachi 70P-72, Tokyo, Japan). The supernatant of the ultracentrifuged samples was transferred into clean tubes and centrifuged at 100,000 × *g* for 60 min. The precipitation was then suspended with Tris-HCl buffer (pH 7.4), and centrifuged at 100,000 × *g* for 60 min. At last precipitation was suspended with Tris-HCl buffer by sonicating (Sonics & Materials, Newtown, CT, USA) 10 s for 6 times on ice, the microsome samples were immediately frozen in liquid nitrogen and stored at −80 °C.

### 3.4. Rat Nuclear Protein Extraction

Livers were homogenized in ice-cold buffer (10 mM HEPES, 1.5 mM MgCl_2_, 10 mM KCl, 0.5 mM DTT, 0.2 mM PMSF, 1 μg/mL pepstatin, 5 μg/mL leupeptin, 10 μg/mL aprotinin, and 0.1% Nonidet P-40) and centrifuged at 3,000 × *g* for 20 min (Anke TGL-16G, Shanghai Anting Scientific Instrument Factory, Shanghai, China) at 4 °C. Pellets were incubated on ice for 0.5 h in high salt buffer and centrifuged at 14,000 × *g* at 4 °C for 20 min. The supernatant was dialyzed for 2 h against nuclear protein dissolution (20 mM HEPES, 400 mM NaCl, 1.5 mM MgCl_2_, 1.2 mM EDTA, 0.5 mM DTT, 0.2 mM PMSF, 1 μg/mL pepstatin, 5 μg/mL leupeptin, 10 μg/mL aprotinin, and 25% glycerol). The dialysate was centrifuged at 14,000 × *g* at 4 °C for 20 min, and the supernatant as collected and stored at −70 °C.

### 3.5. CYP450 Contents and CYP3A1/2 Activities

The protein concentration was determined by BCA assay kit. The contents of CYP450 were measured as described by Omura and Sato [24]. N-Demethylase activities were commonly used to represent the activity of CYP3A [16]. Using an incubation mixture (in a total volume of 1.0 mL) contained liver microsomes (about 0.5 mg protein), 0.05 mmol/L EDTA, 100 mmol/L phosphate buffer (pH 7.4), a substrate (0.4 mmol/L erythromycin) and 1 mmol/L NADPH, the activity of CYP3A1/2 activities were assayed exactly. The reaction between CYP 3A1/2 in liver microsome proteins and substrate erythromycin was started by adding the NADPH after 2 min preincubation at 37 °C. The reaction was run for 20 min at 37 °C and terminated by adding 50 µL ZnSO_4_ (25%). Proteins in this reaction system were precipitated using 50 µL Ba(OH)_2_ saturated solution. Then the mixture of proteins and Ba(OH)_2_ was centrifuged at 2,500 × *g* for 5 min (Anke TGL-16G) at room temperature. The supernatant was decanted into eppendorf tube and incubated with 0.15 mL Nash reagent (containing 1.3 mol/L ammonium acetate, 0.67% acetyl acetone, 3% acetic acid) at 50 °C for 30 min. The absorbance of above samples was detected at 412 nm using UV spectrophotometer (UV 721, Ningbo, China). 

### 3.6. Western Blot Analysis

Liver microsome proteins (25 µg) or liver nuclear proteins (60 µg) were analyzed by sodium dodecyl sulfate-polyacrylamide gel electrophoresis (SDS-PAGE) (12% gel).The separation was performed with constant voltage (80 V per stacking gel and 120 V per running gel) until the dye front reached the bottom edge of the gel. The molecular masses were determined by running pre-stained standard protein markers (Fermentas, Glen Burnie, MD, USA), which covered the range of 10–140 kDa. After fixation in continuous buffer [0.3% (w/v) glycine, 0.6% (w/v) Tris, 0.04% (w/v) SDS, and 20% (v/v) methanol] for 30 min, the proteins in the gel were transferred to nitrocellulose membranes using an electroblotting apparatus (Bio-Rad, Hercules, CA, USA) at 20 V for 1 h. Membranes of CYP3A1/2 and PXR were then blocked with 5% skim milk in TBST buffer [0.2 M Tris-HCl buffer (pH 8.0), 0.05% (v/v) Tween 20, 0.1 M NaCl] for 1.5 h at room temperature, while membranes of CAR were blocked with 7.5% (v/v) horse serum for 4 h at temperature. Membranes were incubated with relative antibody [CYP3A1 and CYP3A2 diluted at 1:3000 in TBST, PXR diluted at 1:200 in TBST, CAR diluted at 1:1000 in TBST containing 5% (w/v) bovine serum albumin ] for 2 h at room temperature, and then washed four times for 15 min with TBST buffer. The membranes were subsequently treated with HRP-conjugated anti-rabbit secondary antibodies at 1:3000 in TBST for 1 h at room temperature followed by washing 15 min for four times with TBST buffer. Immunoreactive proteins were visualized with an enhanced chemiluminescence (eECL) Western blotting detection reagent and captured on X-ray film. The control samples were also analyzed. Blots were reprobed with antibody to β-actin (1:2000) as a loading control. The lanes were scanned (300 dpi resolution) with a Scan Maker 8700 (Microtek, Shanghai, China). Quantitative analysis of immunoblotted bands was performed by Quantity One (Bio Rad).

### 3.7. Quantitative Reverse Transcription-Polymerase Chain Reaction

Total RNA was isolated from the rat livers using RNAiso Plus (Takara, Dalian, China) according to the manufacturer’s protocol. The RNA pellet was washed with 70% ethanol and dissolved into diethyl pyrocarbonate (DEPC) water, and then stored at −70 °C. cDNA was synthesized from 1 μg of total RNA in a total volume of 20 μL using the PrimeScript Reverse Transcription reagent kit (with RNA Eraser) and primer (Takara). The levels of gene expression in liver tissues were analyzed by fluorescent quantitation PCR. The primers and PCR conditions used for CYP3A1/2 and GAPDH amplifications were as described below (Table 2). The reaction specificity was confirmed by melting curve analysis. GAPDH was used as a housekeeping gene for normalization. PCR was performed using SYBR Premix Ex Taq^TM^ (Takara) according to the manufacturer’s instructions in a reaction volume of 20 μL. Reaction mixtures for quantification contained 2 μL RT product, 0.4 μM of each primer, 0.4 μL ROX Reference DyeⅡ and 10 μL SYBR Premix Ex Taq^TM^Ⅱ. The quantitative PCR was monitored by measuring the increase in fluorescence by the binding of SYBR to the generated double-stranded cDNA. All quantitative PCR reactions were performed in duplicate. 

**Table 2:** Sequences of primers used for the FQ-PCR analysis and PCR conditions.

Isoforms	Primer sequence	Product size(bp)	PCR conditions		
			Cycle	Denaturation	Annealing
CYP3A1	F GGA AAT TCG ATG TGG AGT GC	328	45	95 °C, 5 s	60 °C, 34 s
	R AGG TTT GCC TTT CTC TTG CC				
CYP3A2	F AGT AGT GAC GAT TCC AAC ATA T	252	45	95 °C, 5 s	60 °C, 34 s
	R TCA GAG GTA TCT GTG TTT CCT				
PXR	F GAC GGC AGC ATC TGG AAC TAC	112	40	95 °C, 5 s	59 °C, 34 s
	R TGA TGA CGC CCT TGA ACA TG				
CAR	F CAG CCT GCA GGT TGC AGA AG	410	45	95 °C, 5 s	66 °C, 34 s
	R TTC CAC AGC CCG CTC CCT TGA				
RXR	F CGC AAA GAC CTG ACC TAC ACC	133	40	95 °C, 5 s	62 °C, 34 s
	R TCC TCC TGC ACA GCT TCC C				
GAPDH	F GTT ACC AGG GCT GCC TTC TC	168	40	95 °C, 5 s	64 °C, 34 s
	R GGG TTT CCC GTT GAT GAC C				

### 3.8. Biological Accumulation of DBDCT in Rat Liver

The inhibition of CYP3A always led to biological accumulation of drugs mainly metabolizing by CYP3A and toxicity. A high-performance liquid chromatograph (HPLC, Waters 2695, Milford, MA, USA) method was developed for the determination of parent drug DBDCT in rat liver. DBDCT was assayed using a Diamonsil C_18_ (4.6 mm × 250 mm, 5 μm) column (Diamonsil, Lake Forest, CA, USA) by isocratic elution with a flow rate of 1.0 mL/min. The mobile phase composition was 0.5% trifluoroacetic acid-methanol (pH = 3.0) (70:30) (v/v) and spectrophotometric detection was carried out at 238 nm at 30 °C.

An atomic fluorescence spectrometry (AFS, AF-610A, Beijing Rayleigh Analytical Instrument Co., Ltd., Beijing, China) was employed to study the biological accumulation of tin (Sn) in rat liver throughout the study. To study whether the metabolites of DBDCT were accumulated in rat liver, AFS was conducted to detect total Sn content in rat liver. A Sn high performance hollow cathode lamp (General Research Institute for Nonferrous Metals, Beijing, China) was used as the radiation source. The lamp current was set to 80 mA and wavelength to 303.4 nm. Quartz tube (7 mm i.d. × 14 mm length, Beijing Rayleigh Analytical Instrument Co., Ltd.) was used as the atomizer, and its temperature was set to room temperature in the experiment. The negative high voltage of photomultiplier was 275 V. In addition, flow rates of carrier gas (Ar) for the AFS instrument was 800 mL·min^−1^. Sn standard solution (1,000 μg/mL) was purchased from Aladdin-reagent (Aladdin, Shanghai, China) and was stored in polyethylene bottle at 4 °C. Working solutions were prepared daily by diluting the stock standard solutions. A 2% (v/v) hydrochloric acid carrier solution was prepared from concentrated hydrochloric acid. All chemicals and reagents used were of analytical-reagent grade or higher purity, and de-ionized water obtained from a Milli-Q Reagent Water System (Canrex Analytic Instrument Co., Ltd., Shanghai, China, 18.2 MΩ·cm^−1^) was used throughout.

### 3.9. Histopathology

The hepatic morphology was assessed using Olympus (Olympus, Hamburg, Germany) light microscopy. The livers from different groups in acute toxicity testing of DBDCT were sliced and fixed in a 10% buffered-neutral formaline solution. The fixed liver tissue slices were processed using Shandon Excelsior automatic tissue processor (Shandon, Cheshire, UK) and embedded in wax with Shandon Histocentre 3 tissue-embedding system (Shandon). The sections were cut to a thickness of 5 μm (Shandon Finesse 325) and subjected to hematoxylin and eosin (H&E) staining, then the slides were subjected to histopathological examination using a microscope attached with a digital camera at a magnification of 20×.

### 3.10. Statistical Analysis

Each experimental procedure was performed at least three times. Data were expressed as the mean ± SD and performed using the Student *t*-test. A *p*-value less than 0.05 was considered statistically significant.

## 4. Conclusions

In summary, this study leads to several important conclusions. First, CYP3A was inhibited by DBDCT through the down-regulation of its activity, protein and mRNA levels. Second, CAR can mediate the down-regulation of CYP3A by DBDCT. Third, Sn accumulated in rats treated with DBDCT and this accumulation may be caused by inhibition of CYP3A. The inhibition of CYP3A and the biological accumulation of Sn in rat liver is the probable toxic mechanism of DBDCT and some other organotin(IV) compounds.
